# Cultured epidermal stem cells in regenerative medicine

**DOI:** 10.1186/s13287-017-0587-1

**Published:** 2017-07-04

**Authors:** Catherine J. Jackson, Kim Alexander Tønseth, Tor Paaske Utheim

**Affiliations:** 1 Department of Medical Biochemistry, Oslo University Hospital, Oslo, Norway; 2 Department of Plastic Surgery, Oslo University Hospital, Oslo, Norway; 3 Institute of Oral biology, Faculty of Dentistry, University of Oslo, Oslo, Norway; 4 Institute of Clinical Medicine, Faculty of Medicine, University of Oslo, Oslo, Norway; 5 Department of Ophthalmology, Oslo University Hospital, Oslo, Norway

**Keywords:** Regenerative medicine, Stem cells, Burns, Skin wounds, Scars, Chronic wounds, Epidermolysis bullosa, Eye, Limbal stem cell deficiency, Urethra, Plastic surgery, Vitiligo

## Abstract

Transplantation of cultured epidermal cell sheets (CES) has long been used to treat patients with burns, chronic wounds, and stable vitiligo. In patients with large area burns this can be a life-saving procedure. The ultimate goal, however, is to restore all normal functions of the skin and prevent scar formation. Increased focus on the incorporation of epidermal stem cells (EpiSCs) within CES transplants may ultimately prove to be key to achieving this. Transplanted EpiSCs contribute to restoring the complete epidermis and provide long-term renewal.

Maintenance of the regenerative potential of EpiSCs is anchorage-dependent. The extracellular matrix (ECM) provides physical cues that are interpreted by EpiSCs and reciprocal signaling between cells and ECM are integrated to determine cell fate. Thus, the carrier scaffold chosen for culture and transplant influences maintenance of EpiSC phenotype and may enhance or detract from regenerative healing following transfer.

Long-term effectiveness and safety of genetically modified EpiSCs to correct the severe skin blistering disease epidermolysis bullosa has been shown clinically. Furthermore, skin is gaining interest as an easily accessible source of adult epithelial stem cells potentially useful for restoration of other types of epithelia. This review highlights the role of EpiSCs in the current treatment of skin injury and disease, as well as their potential in novel regenerative medicine applications involving other epithelia.

## Background

Cultured epidermal cell sheets (CES) were first used clinically to treat large area burns in 1984, saving the lives of two young brothers [1]. While their value in this life-saving procedure cannot be disputed, normal function and appearance of transplanted areas can be improved. Various skin substitutes have been developed over the last decades with the aim of improving function, strength, and integration of transplanted CES. However, the question of quality of cells used to populate these constructs has received less focus. Evidence suggests that enrichment for epidermal stem cells (EpiSCs) within CES is one factor that may improve outcome/function [2], decrease scar formation [3] and provide long-term regeneration [4]. Beyond this, EpiSCs represent an easily accessible source of autologous adult stem cells (SCs) that may hold great potential for regeneration of other epithelia in the body. This review covers the use of EpiSCs in treatment of skin injury and disease as well as their potential for use in regeneration of other epithelia.

## Biology of skin and wound healing

The skin is composed of two layers, the epidermis and dermis, separated by a thin basement membrane composed of specialized extracellular matrix (ECM) proteins. It acts as a shield against mechanical forces, pathogens, and UV radiation and contributes to systemic homeostasis by maintaining temperature, hydration, and salt levels. It is a labile tissue; the epidermis is renewed approximately every 4 weeks. Self-renewing EpiSCs [5] are largely quiescent in vivo, but are highly proliferative and form large clones in culture [6]. Their progeny, transient amplifying (TA) cells, divide to maintain epidermal homeostasis before themselves committing to terminal differentiation. Skin contains separate SC compartments located in niches found on the basement membrane of the interfollicular epidermis, in the bulge region of the hair follicle, and in the sebaceous gland [7]. Each maintains their own discreet cell number though they have been shown to supplement each other’s populations under certain conditions [8].

**Fig. 1:**
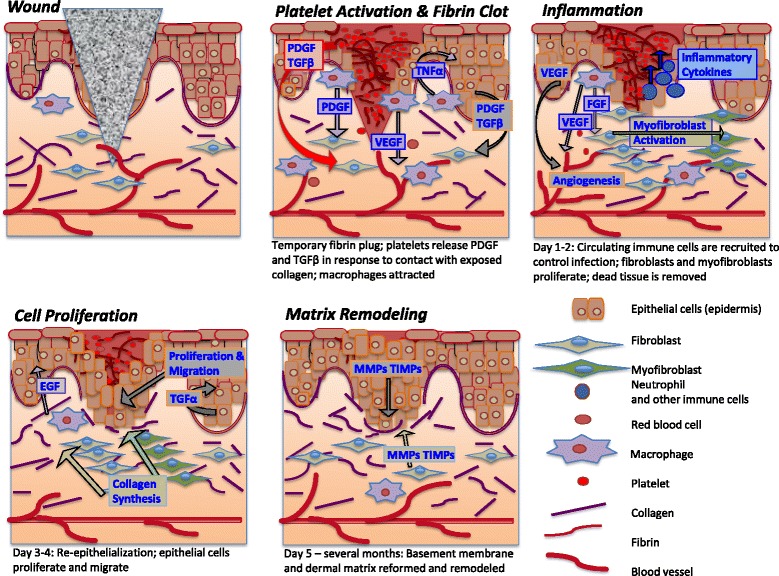
Spatiotemporal coordination of wound healing. *PDGF* platelet-derived growth factor, *TGFβ* transforming growth factor beta, *TNFα* tumor necrosis factor alpha, *VEGF* vascular endothelial growth factor, *FGF* fibroblast growth factor, *EGF* epidermal growth factor, *MMP* matrix metalloproteinase, *TIMPS* tissue inhibitors of MMPs

Wound healing occurs in a highly organized sequence of phases: inflammation, cell proliferation, and matrix remodeling (Fig. 1). During the inflammatory phase the wound is sealed by fibrin, which acts as a temporary matrix. Circulating immune cells invade the new matrix, remove dead tissue, and control infection. Proliferating fibroblasts are recruited and secrete collagen to form granulation tissue and angiogenesis is promoted. Myofibroblasts derived from local fibroblasts express α-smooth muscle actin and contract the wound area. Re-epithelialization occurs as local SC populations are mobilized to proliferate and migrate from the edge of the wound. The specific contribution of interfollicular keratinocytes to wound healing has been illustrated by their extended recruitment in wounds in the absence of hair follicle SCs in a hairless mouse model [9]. In the final phase, fibroblasts and epidermal keratinocytes secrete new ECM components and remodel the matrix through secretion of matrix metalloproteinases (MMPs). Regenerated skin regains 80% of the strength of normal skin at 3 to 4 months post-injury.

## Extracellular matrix

The dermis provides structure and cushioning against mechanical injury. Rather than providing static support, dynamic interaction between cells and the ECM affects cell behavior and cell fate. Secretion of ECM and remodeling factors, matrix MMPs, occurs continually both in vivo and in vitro. Conversely, remodeled ECM can affect cell behavior by exposure of hidden cryptic cell signaling sites that have been enzymatically processed to promote cell migration [10].

The ECM also promotes and extends signaling by acting as a slow release reservoir and delaying growth factor degradation [11]. Corralling of growth factors through ECM modification alters their solubility and bioavailability. Growth factor capture and oligomerization lead to more effective presentation to cell surface receptors.

## Maintenance of epidermal stem cell potency and wound healing

The basement membrane is fundamental in determining EpiSC fate in terms of differentiation status and potency. As with other SC populations in the body, loss of contact with the specialized niche of the basement membrane results in loss of EpiSC clonogenicity. Adhesion is maintained via integrin-β1, which activates a non-differentiation signal in EpiSCs [12]. A sufficient number of integrins must be activated for EpiSCs to remain in the niche [13].

In vivo work has demonstrated the importance of integrin-β1 in wound healing, especially during early re-epithelialization. In mice with keratinocyte-specific deletion of integrin-β1 proliferation is maintained, but migration is impaired and cells accumulate at the wound edge [14]. Though wounds eventually heal they have a changed, flattened, smooth appearance, suggesting that re-epithelialization occurs through a compensatory mechanism in the absence of integrin-β1.

Activation of integrin-β1 is thus indispensable for maintenance of EpiSCs and re-epithelialization following injury. It is therefore important that the substrate selected for culture and transfer of CES maintains cell adhesion via integrin-β1 proteins.

## Clinical use of epidermal stem cells

### Benefits of epidermal stem cell enrichment

Long-term renewal function has been shown in transplanted CES by functional testing for the presence of clonogenic SCs in biopsies taken several years later [4]. This is supported by complementary in vitro work and in animal models [15–17]. Conversely, clinical failure of transplants is associated with EpiSC depletion within the transplant [2], which has been attributed to incorrect culture conditions and suboptimal carrier scaffolds.

**Fig. 2:**
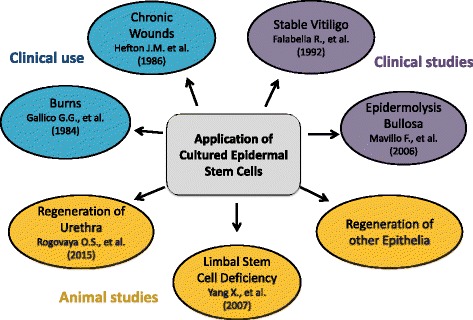
Potential uses of cultured epidermal stem cells in regenerative medicine. Enrichment for epidermal stem cells in cultured epidermal cell sheets could be beneficial in a range of current and novel applications, including: improved outcome in treatment of skin injury, burns, and chronic wounds; gene therapy for epidermolysis bullosa; treatment for stable vitiligo; a source of multipotent adult stem cells for treatment of limbal stem cell deficiency; replacement of urethral epithelium and regeneration of other epithelia in the body

Epidermal SCs have also demonstrated efficacy in the regenerative treatment of other epithelia, such as the cornea in goat [18] and urethra in rabbit [19]. Furthermore, their clonogenicity and long-term persistence can provide lasting treatment for skin diseases such as epidermolysis bullosa (EB) [20] and stable vitiligo [21]. Overall, EpiSCs offer a potential source of autologous clonogenic adult SCs that can be easily harvested for use in diverse regenerative medicine applications (Fig. 2).

### Methods for enrichment of epidermal stem cells

Fluorescence-activated cell sorting (FACS) is a favored technique for SC selection. Sorting for high integrin-β1 expression [22] as well as the combination of high integrin-α6 expression with low transferrin (α6^high^/CD71^low^) [23] have been used for SC enrichment using FACS. Other recently identified cell surface markers associated with EpiSCs include the Notch ligand, Delta-like 1 (DLL1) [24], leucine-rich repeats, and immunoglobulin-like domains 1 (LRIG1) [25] and CD46 [26]. Sorting for cells that maintain export of Hoechst dye is another FACS-based EpiSC sorting technique [15]. Epidermal keratinocyte incubation on collagen IV for 20 minutes is established as a simple method for EpiSC enrichment [22]. Collagen IV favors fast adhesion of EpiSC through their high integrin-β1 expression, whereas more differentiated cells have lower integrin-β1 expression and are rinsed away.

## Skin injury

### Treatment of patients with burns

Transplantation of CES to patients with severe large area burns has been shown to increase patient survival in burn centers throughout the world [1, 27]. However, deficiencies in attachment, integration, appearance, and function remain to be solved [28]. Choice of treatment following debridement of the wound depends on the extent of the injury. In extensive injury less skin is available for split-skin grafts and skin substitutes or CES are used. Skin substitutes are commercial products consisting of a matrix component used with or without cells. They have been criticized for their impermanent nature [29]. Other cited limitations include their simplistic matrix architecture that lacks appropriate mechanical properties that support cell signaling [30]. The next generation of skin substitutes currently under development include composite layers with ECM-compatible form and stiffness, multiple cell types, as well as matrix-bound growth factors [31].

Skin substitutes and CES transplants may be further improved by first enriching for EpiSCs: When the CES culture and transplant are optimal a self-renewing epidermis is formed, indicating EpiSCs are re-established [4]. On the other hand, failure of CES engraftment is associated with low EpiSC content [32]. Besides variation in the number of SCs between patients, the lack of a standard culture protocol to preserve the EpiSC phenotype during culture may be a factor contributing to failure. Enrichment and transfer of EpiSCs may augment and enhance normal wound healing as they retain the capacity to respond to local signaling in a relevant spatiotemporal fashion. Clinical performance may be further improved by use of carrier substrates that mimic the mechanical properties of the SC niche and are compatible with integrin β1 adhesion proteins.

### Excessive wound healing—scarring

Pathological scarring is a major challenge after burns, trauma, and surgery. Scarring results when normal collagen deposition is disrupted by activation of the reparative fibrotic rather than regenerative pathway. Functional tissue is transformed to form a patch of disorganized matrix components and fibroblasts. Scars are characterized by lack of appendages such as hair follicles and sweat glands. Severe scarring results in loss of function, limited movement, restricted growth, adverse aesthetics, and difficult psychological effects. There are two categories of pathological scars; excessive scarring within the original wound borders is referred to as hypertrophic, while keloids are more voluminous and extend beyond the original borders. Scars can also develop contracture by the continued presence of myofibroblasts in the wound.

Remarkably, wounds in early embryos heal without scarring, demonstrating that the genetic program for regeneration must also be present in adults. Furthermore, complete functional and scarless regeneration at specific anatomical sites in mammals, including humans, has been documented post-embryo. For instance, holes in the ears of rabbits completely regenerate within 2 months through a unique mechanism of epidermal down-growths that migrate from the edges to the center of the wound [33]. Amputated digits in mice regenerate through transient formation of a blastema formed from recruited SCs and their re-differentiation to restore original digit morphology [34]. Regeneration of fingertips in children has also been documented [35, 36]. Understanding how this regenerative mechanism of scarless healing is activated has become a major quest.

In scarless embryonic wound healing transforming growth factor beta-1 (TGF-β1) is lower and TGF-β3 is higher [37]. The TGF-β superfamily promotes either repair (TGF-β1 and TGF-β2) or regeneration (TGF-β3) by directing fibroblast behavior and matrix production. However, while administration of TGF-β3 (Avotermin) was shown to be initially promising, the latest clinical trials show no therapeutic effect in scar reduction [3]. Likewise, blocking TGF-β1 and TGF-β2 reduces scarring in animal models, but effects have so far not translated to humans. Wound healing signaling involves intricate spatiotemporal coordination and regulation (Fig. 1). Therefore, a more complex combination of growth factors and/or inhibitors may be necessary to stimulate appropriate regenerative pathways. As well, application of EpiSCs that have higher potency may have greater capacity to proliferate and respond to and initiate signaling related to regeneration.

### Delayed wound healing—chronic wounds

The increased prevalence of diabetes, obesity, vascular disease and an aging population have led to an increase in the number of patients with chronic skin wounds. Complications of non-healing wounds can become serious and require amputation in the most severe cases.

Disruption in normal wound-healing processes can occur due to systemic pathologies that result in local effects such as insufficient blood flow and sustained localized pressure. Similar to treatment of burns, surgical intervention includes application of skin substitutes and CES. A number of commercial skin substitutes have been recommended for chronic ulcers by manufacturers. However, a recent analysis of the usefulness of skin substitutes and grafts in treatment of chronic wounds concluded that while they provide some benefit, strong evidence for their use by evaluation in randomized clinical trials is currently lacking [38].

Chronic wounds lead to compromised local EpiSC populations that become depleted through frequent cycling and yet fail to regenerate the epidermis because of the hostile environment [39]. Inflammation and upregulation of MMPs also prevent normal ECM remodeling and progression of wound healing. Transplantation of CES enriched with EpiSCs on an ECM-compatible carrier substrate may break this negative cycle by simultaneously correcting the EpiSC deficiency and providing matrix components to stabilize the wound site.

## Use of epidermal stem cells in regenerative treatment of other epithelia

The shortage of donor organs has led to increased focus on regenerative medicine to produce replacement tissue and biologically compatible constructs. Autologous EpiSCs may be an ideal alternative source of adult epithelial SCs for use in replacement of damaged epithelia.

When removed from the restrictive in vivo niche environment, EpiSCs demonstrate plasticity beyond their normal cell fate. This has been shown by differentiation of EpiSCs to all three embryonic germ layers following injection into a mouse blastocyst [40]. In another example, cornea-specific cytokeratin expression was seen in epidermal keratinocytes when co-cultured with corneal cells and eye-specific stromal ECM [41].

Skin offers an abundance of easily accessible clonogenic and highly proliferative cells [6]. Moreover, they are especially suited to regeneration of epithelia and do not require complex differentiation protocols as they are of the epithelial lineage.

### Treatment of limbal stem cell deficiency

Injury or loss of limbal SCs (LSCs) that normally maintain homeostasis of the corneal epithelium result in limbal stem cell deficiency (LSCD), a disease that often leads to blindness. A cultured sheet of LSCs taken from the healthy eye can restore the corneal epithelium and a clear cornea [42]. Focus has recently turned to investigation of other epithelial cell types to provide an alternative source of autologous cells.

Cornea and skin epithelia share many similarities, including a typical stratified epithelial morphology and expression of p63, a putative SC marker [43]. While both epithelia are derived from ectoderm, the eye master control gene, PAX6, initiates lens placode invagination during development, delineating ocular epithelial cells from skin epidermal keratinocytes. A key difference is the cytokeratin profile; differentiated epidermal cells express cytokeratin1/10 (CK1/CK10) and corneal epithelial cells express CK3/CK12.

It has been demonstrated that EpiSCs from skin partially or fully restore a clear cornea in eight out of ten eyes in an animal model of LSCD (goats) [18]. The reconstructed corneal epithelium expressed the eye-specific proteins CK3, CK12, and PAX-6 and had stopped expressing skin-specific CK10 by 12 months. In another approach, Ouyang et al. [44] have shown that PAX6-transduced CES are able to maintain a clear cornea in an animal model of LSCD, even after repeated corneal scraping over a long-term period. These studies suggest that despite differences in endogenous cytokeratin expression between skin and cornea, EpiSCs maintain a degree of plasticity and regenerative capacity that may be harnessed for regeneration of other epithelia, with or without prior genetic modification.

### Regeneration of urethra

While skin grafts have been used to restore urethral function and can adapt to the harsh environment of urine exposure, complications arise from hair growth in the urethral lumen in later years [19]. Use of cultured urethral epithelium from the bladder shows promise as an alternative regenerative approach, but it involves an invasive procedure and an additional area of injury [45]. Culture and transplant of CES addresses both of these shortcomings; it supplies epithelial cells that do not grow hair and accommodates the need for epithelial cells harvested from an easily accessible area of the body.

Using a rabbit model of urethral injury, Rogovaya et al. [19] demonstrated successful restoration of a functional urethra using transplanted CES. Unassisted urinary function was restored within 4–7 days after surgery and urethra showed no scar or abnormal fistula formation. Long-term persistence of fluorescently labeled EpiSCs was shown and co-localization of urethral marker UP3 was revealed in transplanted cells 45 days after surgery.

### Treatment of stable vitiligo

Vitiligo is a common skin disease that affects 1 to 2% of the world’s population. Achromatic lesions are caused by loss of melanocytes. The disease can enter long periods of quiescence, referred to as stable vitiligo. Surgical treatment options in this stable period include split-skin grafting with or without phototherapy. Grafting often results in a pitted skin surface and does not always improve pigmentation.

Transplantation of a combination of cultured keratinocytes and melanocytes to treat stable vitiligo was first performed in 1992 [21]. Between 60 and 100% re-pigmentation was achieved in five out of nine patients. Several clinical studies have since tested application of CES with various combinations of melanocytes [46]. Expansion of autologous donor cells to produce CES requires a smaller donor biopsy than split-skin grafts, resulting in less scarring at the donor site. Moreover, comparison of CES and non-cultured skin graft transplantation in the same patient has shown similar effectiveness [47]. In 2000, Guerra et al. [48] reported a new approach using CES and a physiological number of melanocytes; 105 achromatic sites were treated in 32 patients. The transplants integrated well with existing skin, color matching was good, and there was no scarring [48]. Long-term follow-up over 12–36 months showed 77% re-pigmentation. Application of CES without melanocytes followed by exposure to sunlight was also recently shown to be an effective treatment for localized vitiligo (achieving 50–90% re-pigmentation) [49].

### Treatment of epidermolysis bullosa using gene therapy

Epidermolysis bullosa (EB) is a severe skin disease caused by a genetic mutation in any one of a number of genes involved in making attachments between basal epidermal cells and the basement membrane. It is a devastating and often fatal adhesion disorder in skin.

In a clinical case study of a patient with EB caused by a mutation in the laminin-5 gene, primary EpiSC clones taken from palm biopsies were infected with a retrovirus encoding the corrected version [50]. Nine grafts were transplanted to the patient’s legs. One year later synthesis of normal levels of functional laminin-5 was still observed, together with a normal adherent epidermis in all transplanted areas. Analysis of the regenerated epidermis revealed maintenance by long-lasting, self-renewing transgenic EpiSCs, whereas transduced TA cells were lost soon after grafting. The transplanted areas remained disease-free and had a normal functional epidermis 6.5 years later [20].

## Conclusions

Current treatment of skin wounds using skin grafts, skin substitutes and CES results in skin repair, but comprehensive treatment that emulates the complex regenerative process is lacking. Experimental and clinical evidence points to the potential of transplanted CES enriched with EpiSCs for improved clinical outcome. Development of a carrier scaffold that simulates the niche environment and is compatible with ongoing remodeling processes may maintain the undifferentiated state of EpiSC during culture and following transplantation. Many studies have illustrated that EpiSCs have plasticity and clonogenic properties. Thus, EpiSCs may represent an easily accessible source of adult SCs available for replacement of long-term renewal function not only in skin, but also for regenerative application in other epithelia.

## Abbreviations

CES: Cultured epidermal cell sheet
CK: Cytokeratin
EB: Epidermolysis bullosa
ECM: Extracellular matrix
EpiSC: Epidermal stem cell
FACS: Fluorescence-activated cell sorting
LSC: Limbal stem cell
LSCD: Limbal stem cell deficiency
MMPs: Matrix metalloproteinase
SC: Stem cell
TA: Transit amplifying
TGF-β: Transforming growth factor beta
